# Publisher Correction: Structural insights into chaperone addiction of toxin-antitoxin systems

**DOI:** 10.1038/s41467-019-09110-3

**Published:** 2019-03-07

**Authors:** Valérie Guillet, Patricia Bordes, Cécile Bon, Julien Marcoux, Virginie Gervais, Ambre Julie Sala, Suzana Dos Reis, Nawel Slama, Israel Mares-Mejía, Anne-Marie Cirinesi, Laurent Maveyraud, Pierre Genevaux, Lionel Mourey

**Affiliations:** 1 Institut de Pharmacologie et de Biologie Structurale, IPBS, Université de Toulouse, CNRS, UPS, 31077 Toulouse, France; 2 Laboratoire de Microbiologie et de Génétique Moléculaires, Centre de Biologie Intégrative (CBI), Université de Toulouse, CNRS, UPS, 31062 Toulouse, France

Correction to: *Nature Communications;* 10.1038/s41467-019-08747-4; published online 15 February 2019

The original version of this Article contained errors in Figures 1 and 4. In Fig. 1b, the *Mtb*-SecB^TA^ sequence was displayed incorrectly. In the inset panel within Fig. 4c, the y-axis of the graph incorrectly read (*Q*.R_g_)^2^ × *I*(*Q*)//(0), and should have read (*Q*.*R*_g_)^2^ × *I*(*Q*)/*I*(0). These errors have been corrected in both the PDF and HTML versions of the Article.

