# Supplementary material for: Structural insights into chaperone addiction of toxin-antitoxin systems
Source: Nat Commun. 2019 Feb 15;10:782. doi: 10.1038/s41467-019-08747-4 (PMC6377645; doi:10.1038/s41467-019-08747-4)
Supplement: Supplementary file 1 — Supplementary Information [file 41467_2019_8747_MOESM1_ESM.pdf]

Structural insights into chaperone addiction of toxin-antitoxin systems

V. Guillet et al.

Supplementary Table 1. Tetramer interface characteristics of *Mtb*-SecB^{TA} and SecB.

	Dimer interface A-B			Dimer interface C-D			Dimer-dimer interface A-C			Dimer-dimer interface B-D		
	Interface area (Å^2)	N_{HB}	N_{SB}	Interface area (Å^2)	N_{HB}	N_{SB}	Interface area (Å^2)	N_{HB}	N_{SB}	Interface area (Å^2)	N_{HB}	N_{SB}
Mtb-SecB^{TA}	739	12	11	728	12	12	820	4	0	704	5	1
Ec-SecB (1qyn)	1000	16	7	985	18	7	549	2	0	558	2	0
Hi-SecB (1fx3)	982	14	6	1037	16	5	553	1	0	540	3	0
Ec-SecB-PhoA (5jtl)	987	12	8	1011	12	6	1080	5	0	399	2	2
Hi-SecB-SecAc (1ozb)	1008	17	7	986	15	3	510	1	0	525	2	0

Interfaces were analysed using PDBePISA. Interface areas are calculated as differences in total accessible surface areas of isolated and interfacing structures divided by two. N_{HB} and N_{SB}, number of potential hydrogen bonds and salt bridges across the interface, respectively. All structures have the same tetramer subunit numbering. For 1ozb, there are two tetramers in the asymmetric unit and only one is bound to SecAc, which has been considered here. For 5jtl, the best representative conformer was used.

Supplementary Table 2. Details of *Mtb*-SecB^{TA}-ChAD interactions.

Interface E-A (401 Å^2)[a]			Interface F-D (397 Å^2)			Interface G-C (416 Å^2)		
Chain:Res [Atom]	d (Å)	Chain:Res [Atom]	Chain:Res [Atom]	d (Å)	Chain:Res [Atom]	Chain:Res [Atom]	d (Å)	Chain:Res [Atom]
E:E104 [N]	3.7	A:D60 [OD2]						
						G:**P106** [O]	2.8-2.8	C:**R29** [NH2]
E:**P106** [O]	3.8	A:**R29** [NH2]				G:**P106** [O]	3.6	C:**R29** [NH2]
E:**W108** [NE1]	2.7	A:**D27** [OD2]	F:**W108** [NE1]	2.8	D:**D27** [OD2]	G:**W108** [NE1]	2.8	C:**D27** [OD2]
						G:**W108** [O]	2.7-2.8	C:**D27** [OD2]
E:R110 [NE]	3.5	A:**D27** [OD1]	F:R110 [NE]	3.4	D:**D27** [OD1]	G:R110 [NE]	3.5	C:**D27** [OD1]
E:R110 [NE]	3.2	A:**D27** [OD2]	F:R110 [NE]	3.2	D:**D27** [OD2]	G:R110 [NE]	2.8	C:**D27** [OD2]
E:R110 [NH2]	3.2	A:**D27** [OD1]	F:R110 [NH2]	3.1	D:**D27** [OD1]	G:R110 [NH2]	3.1	C:**D27** [OD1]
E:R110 [NH2]	3.5	A:**D27** [OD2]	F:R110 [NH2]	3.6	D:**D27** [OD2]	G:R110 [NH2]	3.4	C:**D27** [OD2]
E:R110 [NH2]	2.9	A:I28 [O]	F:R110 [NH2]	2.8	D:I28 [O]			
Residues above + E:**V105**, **T107**	VDW[b]	A:**D58**, A59, T63, I64, **A66**, **F67**, **V68**, R70, **A106**, **L108**	Residues above + F:E104, **V105**, **P106**, **T107**	VDW	D:**R29**, L31, **D58**, A59, P60, **I64**, A66, **F67**, **V68**, R70, **A106**, **L108**	Residues above + G:E104, **V105**, **T107**, H109, R115	VDW	C:R26, I28, L31, **D58**, A59, D60, I64, A66, **F67**, **V68**, R70, **A106**, **A107**, **L108**
Interface E-C (322 Å^2)			Interface F-B (323 Å^2)			Interface G-A (322 Å^2)		
						G:R110 [NH2]	3.9	A:Y146 [OH]
E:S112 [OG]	2.8-2.9[c]	C:**T158** [N]						
E:**S113** [N]	2.9-2.7	C:P156 [O]	F:**S113** [O]	3.2	B:**L47** [N]	G:S113 [O]	2.8	A:**L47** [N]
E:**Y114** [OH]	2.6	C:**P155** [O]	F:**Y114** [OH]	2.5	B:**P155** [O]	G:**Y114** [OH]	2.7	A:**P155** [O]
E:R115 [N]	2.9	C:**L47** [O]	F:R115 [N]	3.1	B:**L47** [O]			
E:R115 [O]	2.9	C:**T48** [OG1]	F:R115 [O]	3.8	B:Y49 [N]			
Residues above + E:R110	VDW	C: P43, **G46**, Y49, **I77**, Q80, R142, L154, **L157**, I161	Residues above + F:R110, S112	VDW	B:**T48**, **I77**, Q80, R142, L154, P156, **L157**, **T158**	Residues above + G:**S112**, R115	VDW	A:K42, **G46**, T48, Y49, **I77**, Q80, T149, L154, **P156**, **L157**, T158
Interface E-B (128 Å^2)			Interface F-C (128 Å^2)			Interface G-D (201 Å^2)		
E:R110 [NH1]	3.0	B:G150 [O]	F:R110 [NH1]	3.0	C:Gly150 [O]			
Residue above + E:W108, H109, L111	VDW	B:A40, **P41**, K42, R151, **A153**, L154, P155, P156	Residue above + F:W108, H109, L111	VDW	C:P41, P43, R151, **A153**, L154, P155, P156	G:T107, W108, H109, R110, L111	VDW	D:**A40**, P41, **P43**, A44, G150, **A153**, **L154**

[a] Each ChAD peptide (chains E, F, and G corresponding to sequence $_{104}$EVPTWHRLSSYRG$_{116}$ of *Mtb*-HigA1)) interacts with three of the four *Mtb*-SecB^{TA} subunits (A, B, C, and D). For each interface, the interface area and polar interactions (chain identifier, residue, atom, and distance between bridging atoms) are detailed. [b] VDW, van der Waals interactions (only listed for residues not involved in polar interactions). [c] When atoms are bridged by a water molecule, the two corresponding distances are given. Residues in bold have ≥ 50% of their surface area buried upon interactions (within a given interface). Equivalent interactions are on a same line.

Supplementary Table 3. List of primers used for QuickChange mutagenesis.

Mutant	Primer name	Primer sequence
D27A	Rv D27A FOR<br>Rv D27A REV	cgcacagatccgcgcgatccggctgctgcg<br>cgcagcagccggatcgcgcggatctgtgcg
I28A	Rv I28A FOR<br>Rv I28A REV	cagatccgcgatgcgcggctgctgcgc<br>gcgcagcagccgcgcatcgcggatctg
R29A	Rv R29A FOR<br>Rv R29A REV	cagatccgcgatatcgcgctgctgcgcactcag<br>ctgagtgcgcagcagcgcgatatcgcggatctg
P41A	Rv P41A FOR<br>Rv P41A REV	ctgtccatcgtgcggcgaagcctgcgcaggg<br>ccctgcgcaggcttcgccgcacgatggacag
P43A	Rv P43A FOR<br>Rv P43A REV	catcgtgcgcccaaggcggcgcagggcctgac<br>gtcaggccctgcgccgccttgggcgcacgatg
G46A	Rv G46A FOR<br>Rv G46A REV	caagcctgcgcaggcgctgacctacgacctc<br>gaggtcgtaggtcagcgcctgcgcaggcttg
L47A	Rv L47A FOR<br>Rv L47A REV	caagcctgcgcagggcgcgacctacgacctcgag<br>ctcgaggtcgtaggtcgcgccctgcgcaggcttg
T48A	Rv T48A FOR<br>Rv T48A REV	ctgcgcagggcctggcgtacgacctcgagttc<br>gaactcgaggtcgtacgccaggccctgcgcag
Y49A	Rv Y49A FOR<br>Rv Y49A REV	gcgcagggcctgaccgcggacctcgagttcgaac<br>gttcgaactcgaggtccgcggtcaggccctgcgc
D58A	Rv D58A FOR<br>Rv D58A REV	gaacccgctgtggcggccgatccggccac<br>gtggccggatcggccgccacagcgggttc
D60A	Rv D60A FOR<br>Rv D60A REV	ccgctgtggatgccgcgccggccactatctc<br>gagatagtggccggcgcggcatccacagcgg
I64A	Rv I64A FOR<br>Rv I64A REV	ccgatccggccactgcgtcagcatttgtggtg<br>caccacaaatgctgacgcagtggccggatcgg
F67A	Rv F67A FOR<br>Rv F67A REV	ccactatctcagcagcggtggtgcggatttc<br>gaaatccgcaccaccgctgctgagatagtgg
V68A	Rv V68A FOR<br>Rv V68A REV	cactatctcagcatttgcggtgcggatttcttgcc<br>ggcaagaaatccgcaccgcaaatgctgagatagtg
I77A	Rv I77A FOR<br>Rv I77A REV	cttgccacctgcgcgcgcaaaaccaggcggcag<br>ctgccgcctggttttgcgcgcgcaggtggcaag
L108A	Rv L108A FOR<br>Rv L108A REV	Gagttcgcggcagcgttcgactaccacttg<br>caagtggtagtcgaacgctgccgcgaactc
G150A	Rv G150A FOR<br>Rv G150A REV	ctacgacctcaccgcgcgtctcgcactgcc<br>ggcagtgcgagacgcgcggtgaggtcgtag
L154A	Rv L154A FOR<br>Rv L154A REV	ccggccgtctcgcagcgccaccgttgaccc<br>gggtcaacggtggcgctgcgagacggccgg
P155A	Rv P155A FOR<br>Rv P155A REV	ggccgtctcgcactggcgccgttgacccttgag<br>ctcaagggtcaacggcgccagtgcgagacggcc
P156A	Rv P156A FOR<br>Rv P156A REV	gtctcgcactgccagcgttgacccttgag<br>ctcaagggtcaacgctggcagtgcgagac
PP155-156AA	Rv PP155-156AA FOR<br>Rv PP155-156AA REV	ccgtctcgcactggcggcgttgacccttgagatattg<br>caatatctcaagggtcaacgccgccagtgcgagacgg
PP155-156GG	Rv PP155-156GG FOR<br>Rv PP155-156GG REV	ccgtctcgcactggggggggttgacccttgagatattg<br>caatatctcaagggtcaacccccccagtgcgagacgg
L157A	Rv L157A FOR<br>Rv L157A REV	gcactgccaccggcgacccttgagatat<br>atatctcaagggtcgccggtggcagtgc
T158	Rv T158 FOR<br>Rv T158 REV	gcactgccaccgttggcccttgagatat<br>atatctcaagggccaacggtggcagtgc

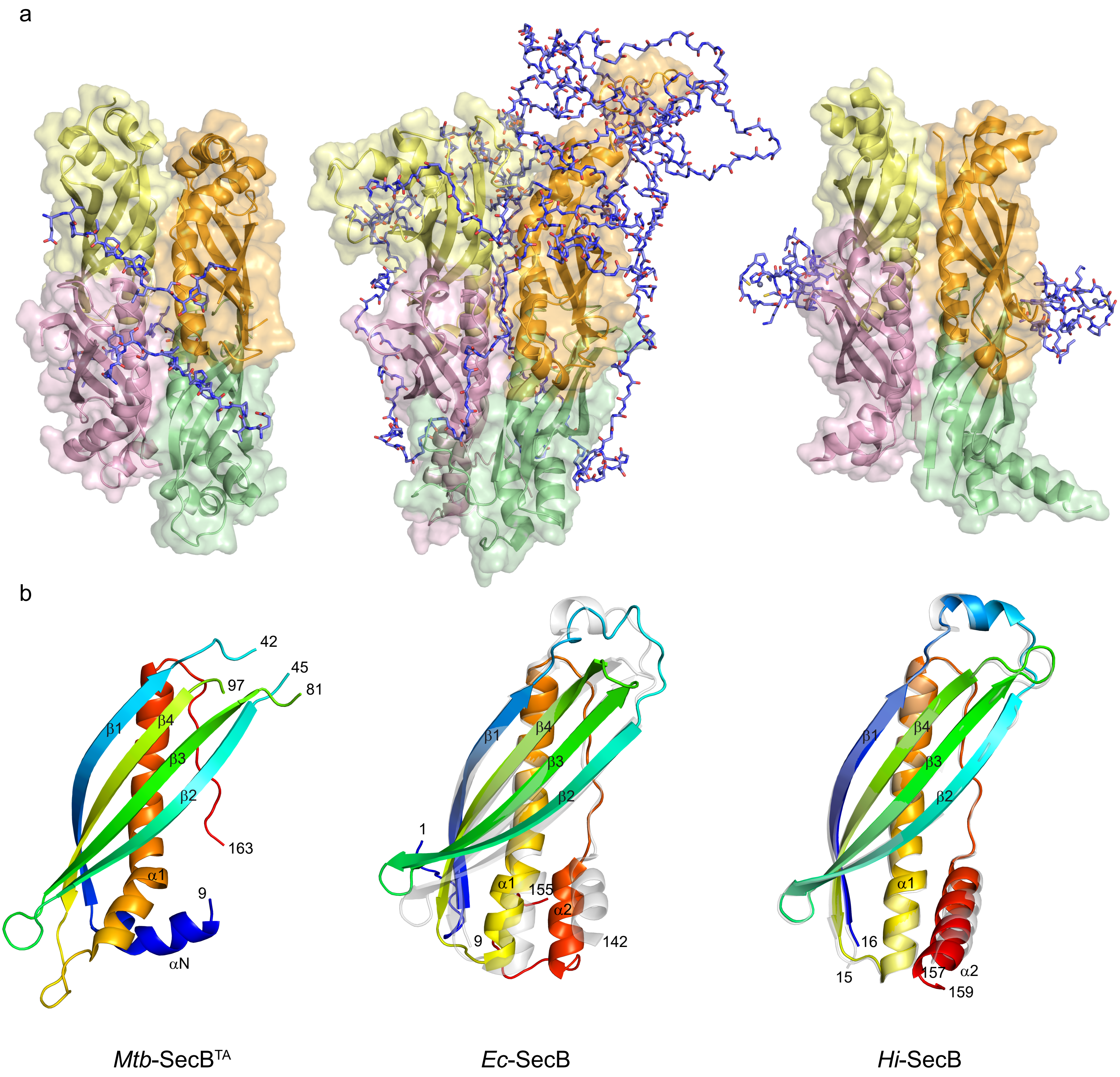

Supplementary Figure 1: *Mtb*-SecB^{TA} and SecB are structurally similar. (a) Ribbon representation and semi-transparent molecular surface of the X-ray structure of *Mtb*-SecB^{TA}/ChAD (this work, left), the NMR structure of *E. coli* SecB in complex with unfolded PhoA (middle, PDB code 5JTL), and the X-ray structure of *H. influenzae* SecB complexed with SecA C terminus (right, PDB code 1OZB). The A, B, C, and D subunits forming the tetramers are respectively colored in green, orange, violet, and yellow whereas the different ligands are in slate blue. For sake of clarity, only the main-chain trace of PhoA has been represented. **(b)** For each structure shown in (a), the fold of subunit A is represented and colored in spectrum mode from blue (N-terminus) to red (C-terminus) with secondary structures labeled and residue boundaries numbered. In addition, the corresponding subunit as found in the crystal structures of free *Ec*-SecB and *Hi*-SecB tetramers (PDB code 1QYN and 1FX3, respectively) is depicted in gray.

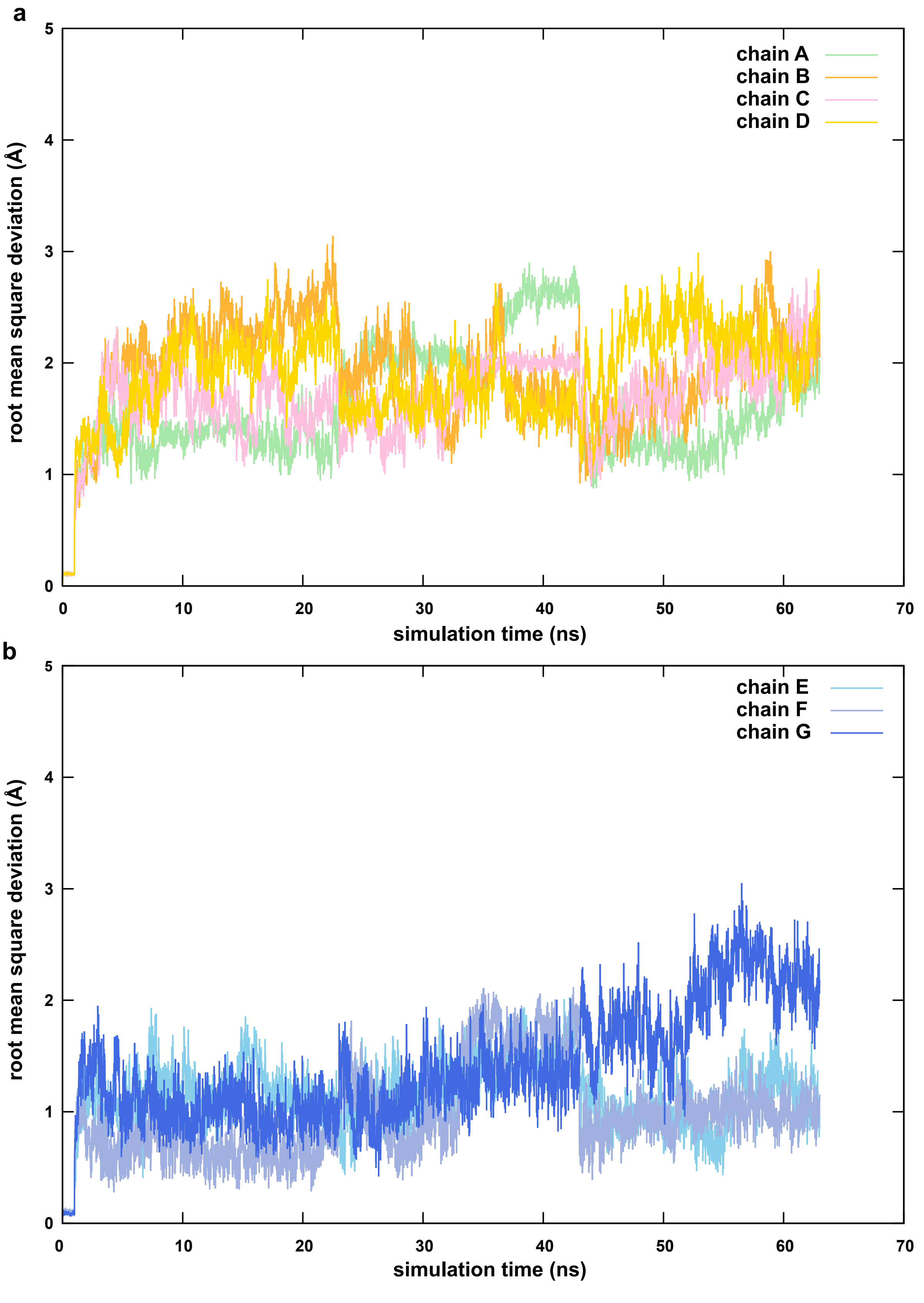

Supplementary Figure 2: Molecular dynamics simulation. The variation of the root mean square deviation (rmsd) of the distance to the crystallographic structure is plotted along the 60 ns simulation. **(a)** rmsd computed independently for each protein chain of the *Mtb*-SecB^{TA} tetramer based on the Cα atoms of residues found in secondary structure elements (see **Fig. 1b**). **(b)** rmsd computed independently for each ChAD peptide using all Cα atoms.

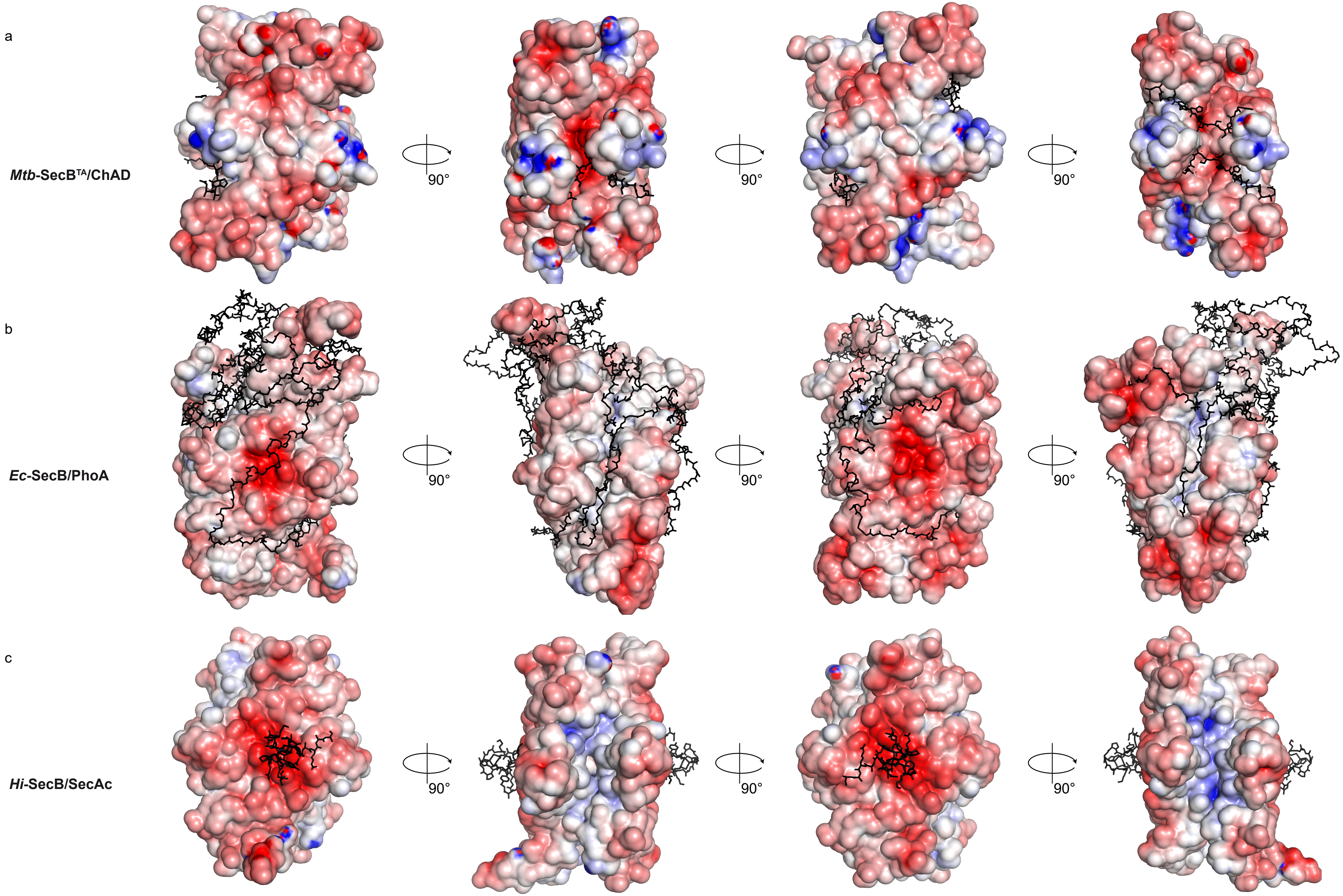

Supplementary Figure 3: *Mtb*-SecB^{TA} and SecB differ in their topographies and electrostatic potential surfaces. Electrostatic potential surface of **(a)** *Mtb*-SecB^{TA}/ChAD, **(b)** *Ec*SecB/PhoA (PDB code 5JTL), **(c)** *Hi*-SecB/SecAc (PDB code 1OZB). Four perpendicular views around a vertical axis are shown. Electrostatic potential was calculated using the protein part of the 3D coordinates and is color-coded from red (-6 $k_B T/e$) to blue (+6 $k_B T/e$); white is neutral. The different ligands are in black. For sake of clarity, only the main-chain trace of PhoA has been represented.

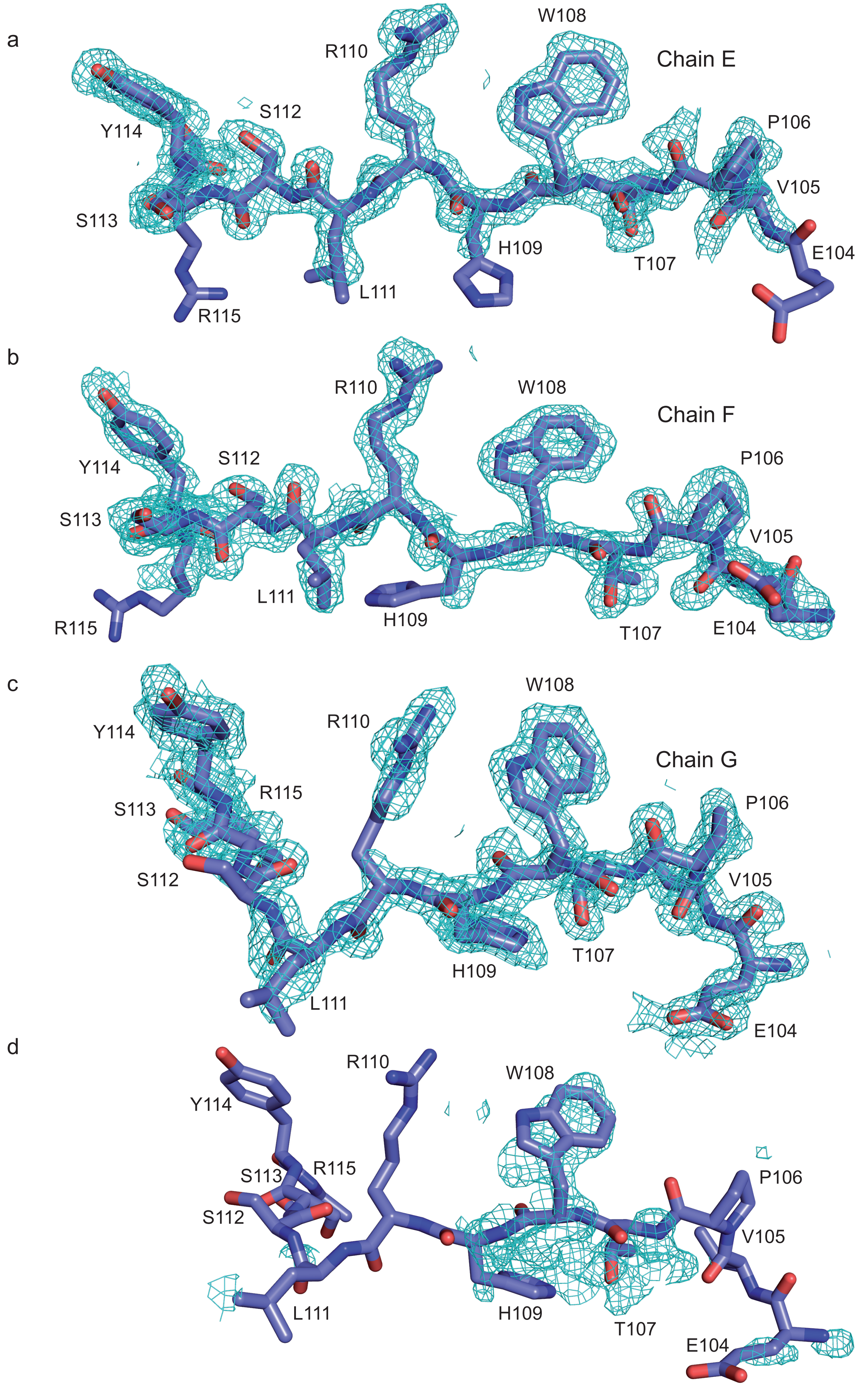

Supplementary Figure 4: Electron density map around the ChAD peptide. The feature enhanced map, calculated after having deleted atoms corresponding to the peptides, contoured at 1.0 sigma is shown in cyan. **(a)** Chain E. **(b)** Chain F. **(c)** Chain G. **(d)** Around what would be a fourth peptide-binding site where a 2-fold symmetry mate of peptide G has been generated through the DCBA permutation.

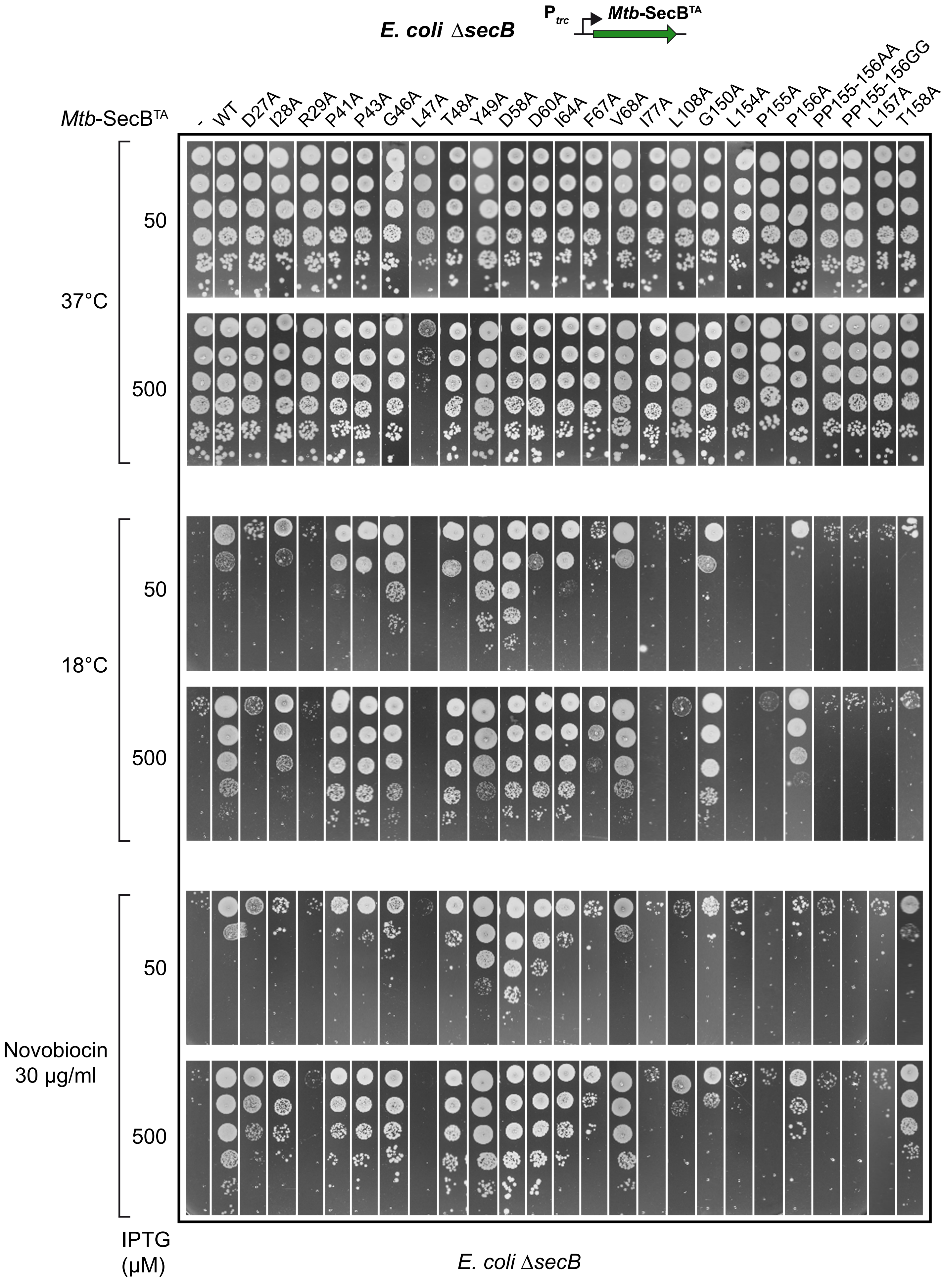

Supplementary Figure 5: Generic chaperone functions of TA-directed *Mtb*-SecB^{TA} variants. W3110 Δ*secB* strain transformed with p29-*Mtb*-SecB^{TA} or its mutant derivatives was grown at 37 °C to mid-log phase in LB ampicillin, serially diluted, and spotted on LB ampicillin agar plates in the absence or presence of IPTG inducer. Plates were incubated overnight at 37 °C or for 3 days at 18 °C. For novobiocin resistance assay, 30 µg ml^{-1} of novobiocin was added and plates were incubated overnight at 37 °C.

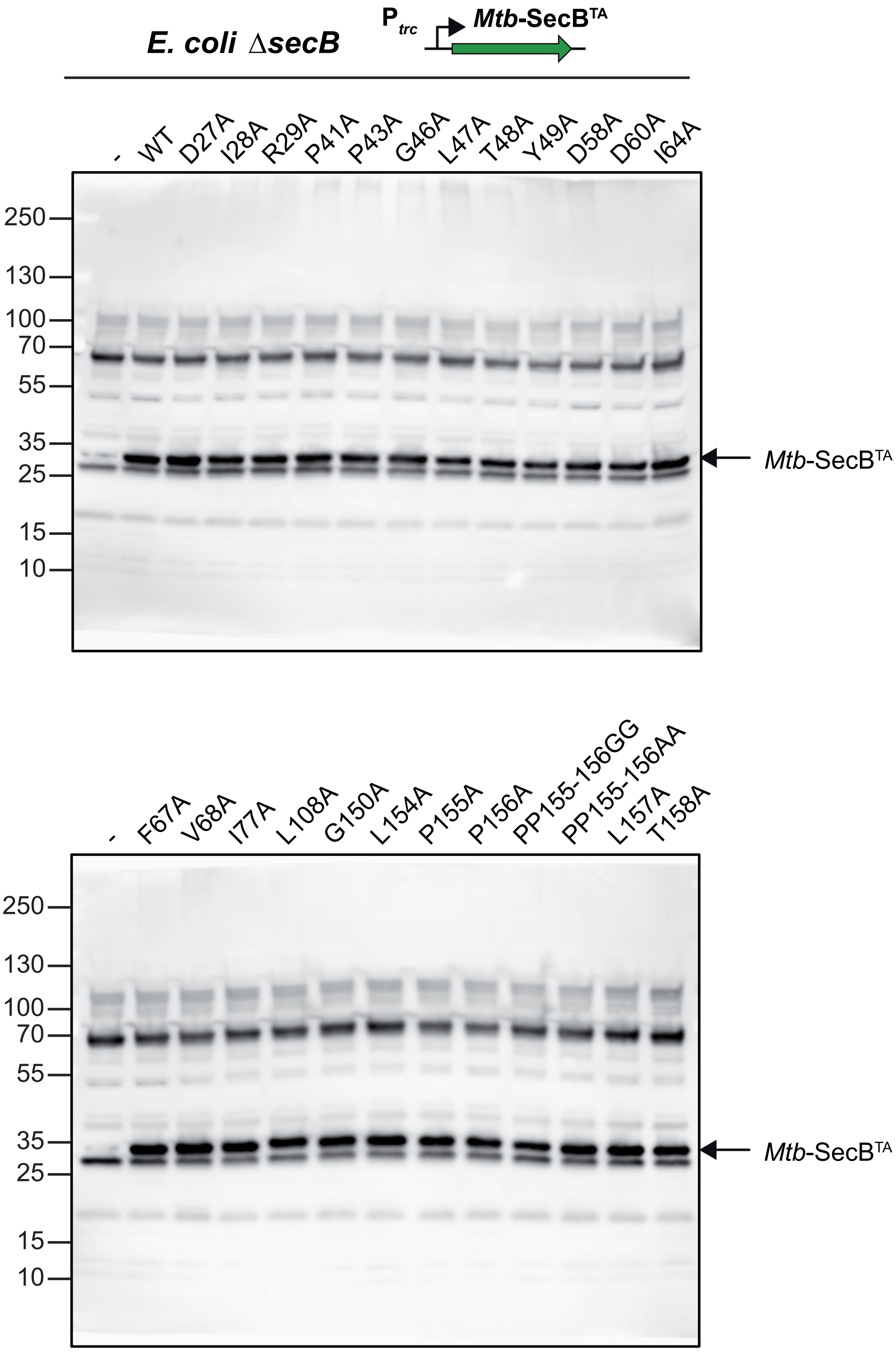

Supplementary Figure 6: Expression of the *Mtb*-SecB^{TA} mutants. Transformants of strain W3110 *ΔsecB* containing p29SEN-*Mtb*-SecB^{TA} mutant derivatives were grown at 37 °C in LB ampicillin. At mid-log phase, IPTG inducer (500 µM) was added 2 h before preparing whole cell extracts. Extracts were separated on mini-PROTEAN 4-15% TGX precast gels (Bio-Rad) and steady state protein expression was visualized following western blotting using anti-*Mtb*-SecB^{TA} antibodies as previously described[1]. Arrows indicate the presence of *Mtb*-SecB^{TA} derivatives.

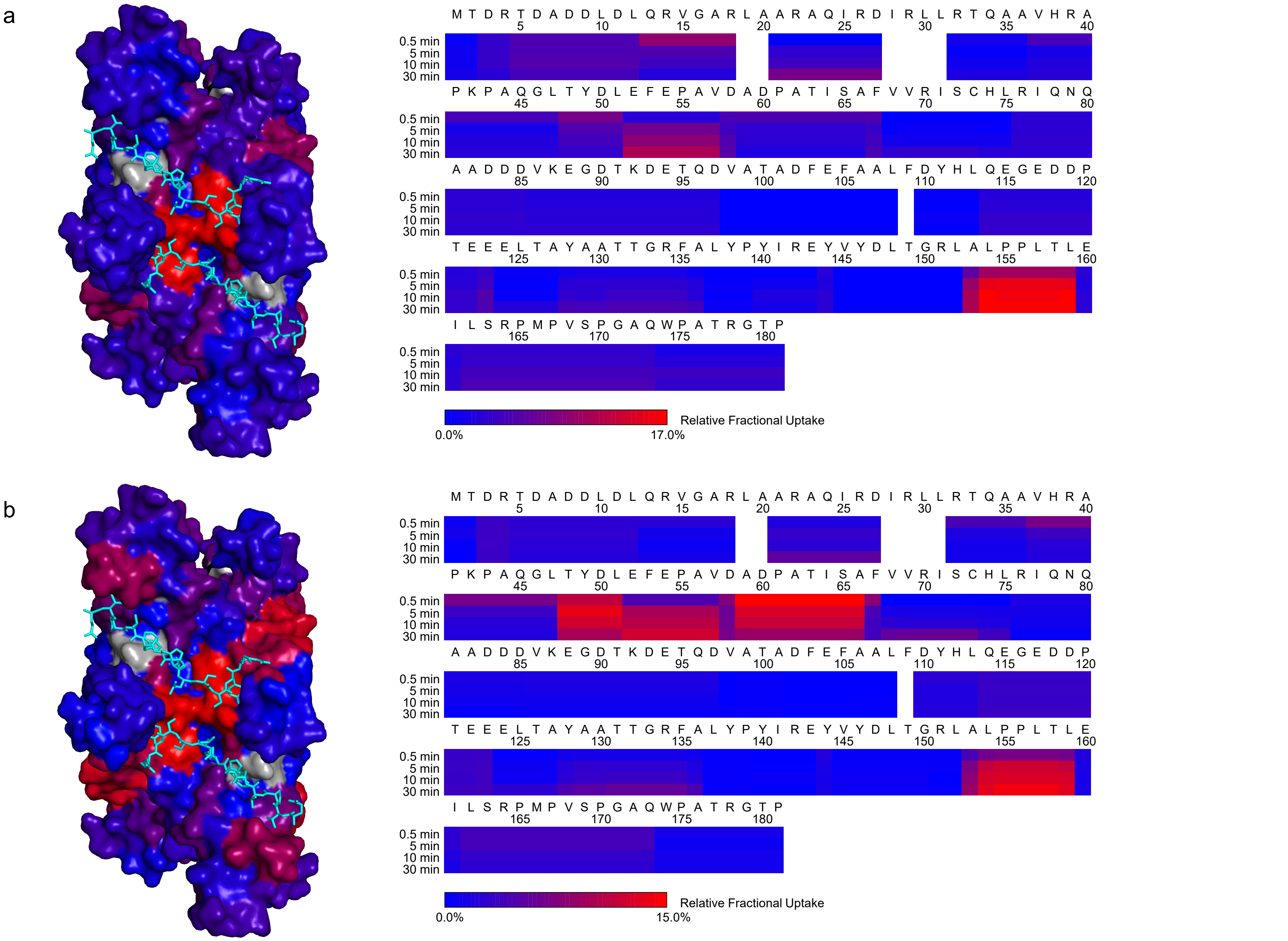

Supplementary Figure 7: Hydrogen Deuterium eXchange MS. Differential hydrogen-deuterium uptake between **(a)** *Mtb*-SecB^{TA} and *Mtb*-SecB^{TA} incubated with 8 molar equivalents of ChAD or between **(b)** *Mtb*-SecB^{TA} and *Mtb*-SecB^{TA}/HigA1. The differential uptake is color-coded from blue (0%) to red (15-17%). Uncovered residues are shown in grey. The results at 30 min deuteration are plotted on the structure of *Mtb*-SecB^{TA}/ChAD complex. The ChAD peptides are represented as sticks.

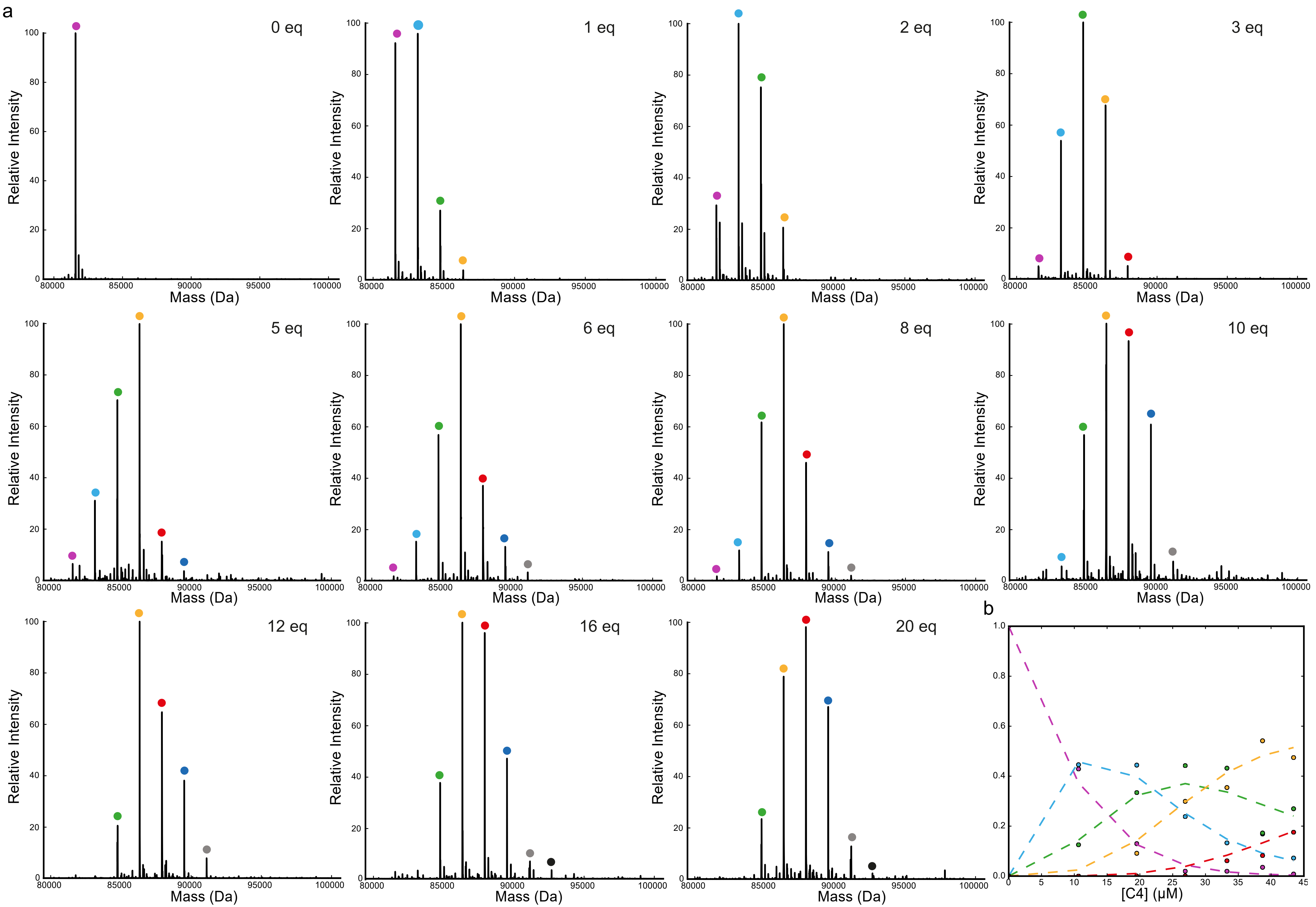

Supplementary Figure 8: Titration of *Mtb*-SecB^{TA} with ChAD peptide. (a) Deconvoluted native mass spectra showing the progressive binding of ChAD to *Mtb*-SecB^{TA}. The species detected correspond to the *Mtb*-SecB^{TA} tetramer (MW 81,575 Da, purple circle) and to the binding of up to seven ChAD peptides with MWs of 83,157; 84,746; 86,334; 87,922; 89,508; 91,094 and 92,685 Da (cyan, green, orange, red, blue, gray, and black circles, respectively). **(b)** Relative abundance of the different observed species upon titration with up to 4 molar equivalents of ChAD. Experimental data (solid circles, same color code) were fitted by the UniDec algorithm (dashed lines).

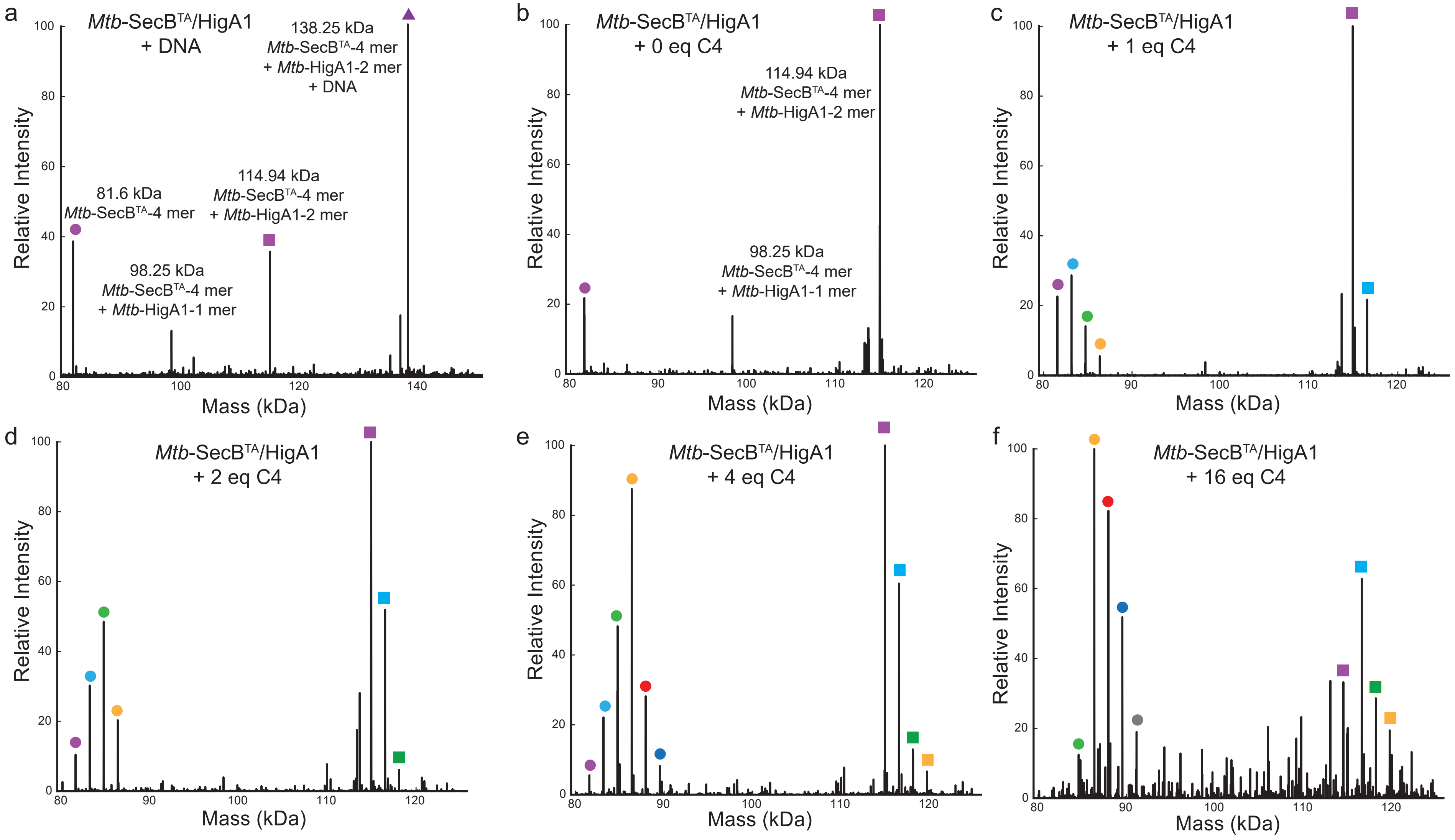

Supplementary Figure 9: DNA-binding properties and disruption by ChAD of *Mtb*-SecB^{TA}/HigA1. (a) Deconvoluted native mass spectrum of *Mtb*-SecB^{TA}/HigA1 in the presence of dsDNA derived from the *higB* P2 promoter. **(b-f)** Deconvoluted native mass spectra of *Mtb*-SecB^{TA}/HigA1 showing how the *Mtb*-SecB^{TA}/HigA1 hexamer is progressively destabilized/dissociated by increasing concentrations of the ChAD peptide. Circles and squares indicate ChAD peptides bound to the *Mtb*-SecB^{TA} tetramer and *Mtb*SecB^{TA}/HigA1 hexamer, respectively (same color code of the symbols as in Supplementary Fig. 8).

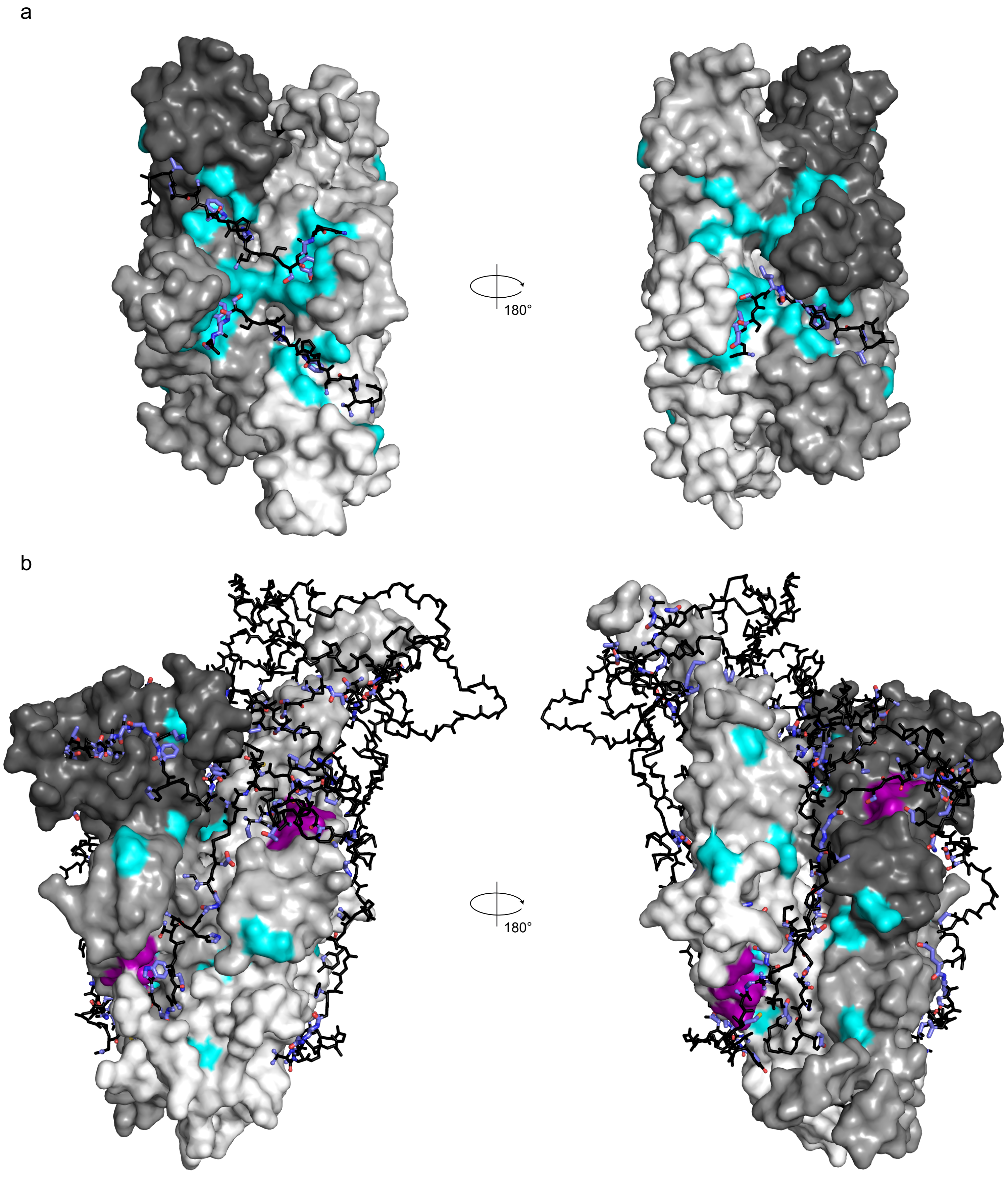

Supplementary Figure 10: Antitoxin binding to *Mtb*-SecB^TA^ is different from client binding to *Ec*-SecB. (a) The molecular surface of the *Mtb*-SecB^TA^ tetramer is represented in variations of grey depicting the four protein subunits. Residues whose ability to maintain TA control were affected upon single alanine mutagenesis are depicted in cyan. The three bound ChAD peptides are shown as black sticks where ChAD atoms found within 4 Å of the protein are shown as enlarged sticks with carbon, nitrogen, and oxygen atoms in slate blue, blue, and red, respectively. Left and right views are related by a 180° rotation around a vertical axis. **(b)** The structure of the *Ec*-SecB/PhoA complex (PDB code 5JTL) is shown in the same orientation, scale and representation scheme. For non-interacting PhoA residues, only the main-chain trace has been represented. Residues V40, L42, and L44 whose triple substitution to alanine causes a 40-fold reduction in the affinity of *Ec*-SecB for PhoA are in purple. Residues whose mutation was found to specifically improve the ability of *Ec*-SecB to control the *Mtb*-HigBA1 TA system without affecting its function in protein export[2] are in cyan.

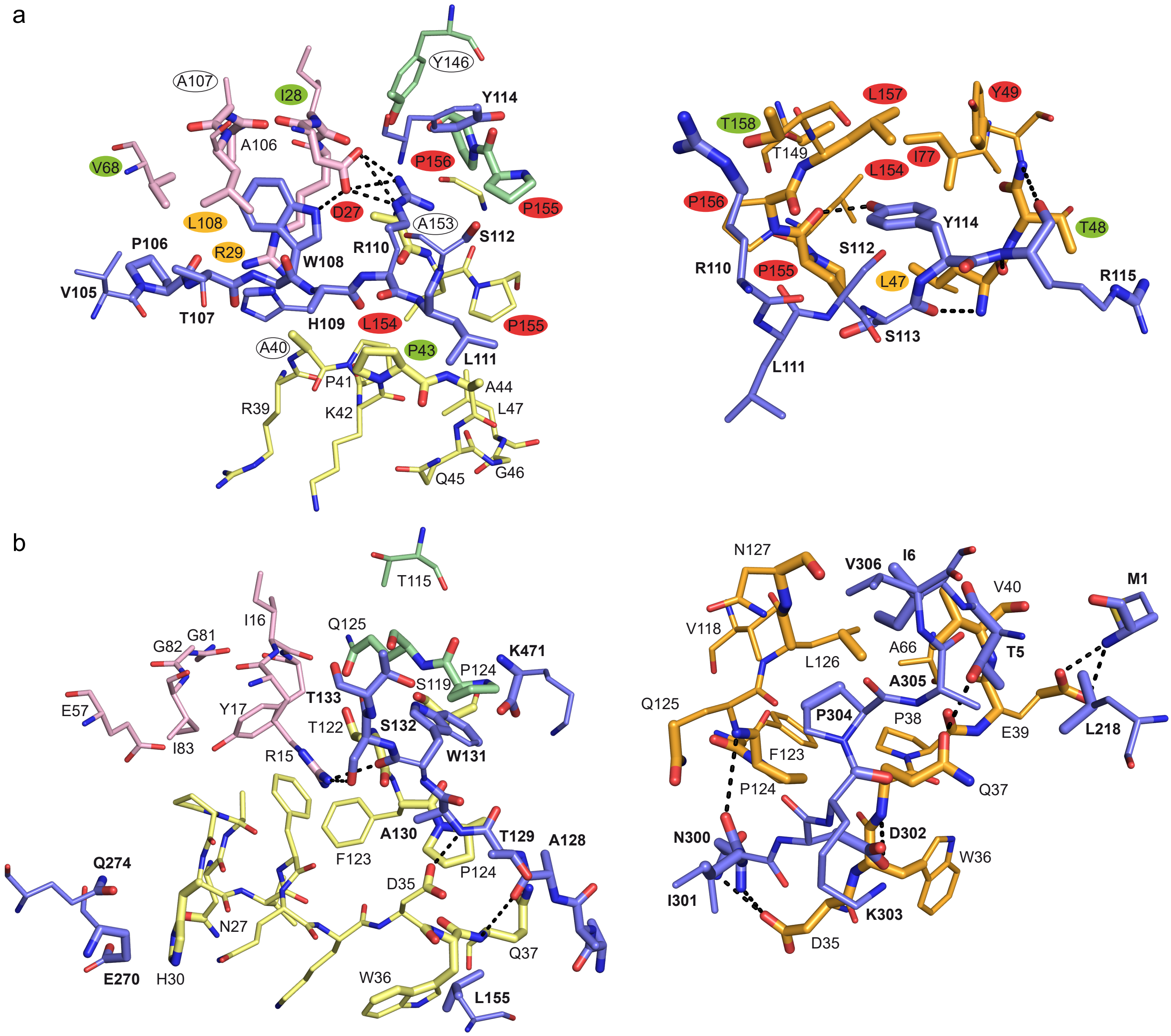

Supplementary Figure 11: *Mtb*-SecB^{TA}/ChAD interaction hot spots have no equivalent in *Ec*-SecB/PhoA. (a) Close-up views centered on ChAD residues W108 and R110 (*Left*, same orientation as in Fig. 2b) and Y114 (*Right*, same orientation as in Fig. 2c). *Mtb*SecB^{TA} atoms found within 5 Å of the peptides are shown as enlarged sticks. Color coding of the labels of residues interacting with ChAD is according to the impact of single alanine scanning on the ability to maintain TA control from green (no effect) to orange (medium effect) to red (most severe effect). Polar interactions are represented by black dotted lines. **(b)** The structure of the *Ec*-SecB/PhoA complex (PDB code 5jtl) is shown in the same orientation, scale and representation scheme.

Supplementary References

1. Bordes, P. *et al.* SecB-like chaperone controls a toxin-antitoxin stress-responsive system in *Mycobacterium tuberculosis*. *Proc Natl Acad Sci U S A* **108,** 8438-8443 (2011).
2. Sala, A.J. *et al.* Directed evolution of SecB chaperones toward toxin-antitoxin systems. *Proc Natl Acad Sci U S A* **114,** 12584-12589 (2017).
