# Supplementary material for: Structural insights into chaperone addiction of toxin-antitoxin systems
Source: Nat Commun. 2019 Feb 15;10:782. doi: 10.1038/s41467-019-08747-4 (PMC6377645; doi:10.1038/s41467-019-08747-4)
Supplement: Supplementary file 3 — Description of Additional Supplementary Files [file 41467_2019_8747_MOESM3_ESM.pdf]

Description of Additional Supplementary Files

File Name: Supplementary Data 1
Description: This zip file contains the DynamX outputs (.pml files) of the deuteration kinetics of Mtb-SecBTA alone, and incubated with the ChaD peptide and HigA1 (Supplementary Figure 7) as well as a .ppt file including the Mtb-SecBTA sequence coverage and the deuteration uptake curves obtained for each peptide in the three conditions.

File Name: Supplementary Data 2
Description: This zip file contains the unprocessed MS spectra corresponding to the titration of Mtb-SecBTA with the ChaD peptide (Figure 4d and Supplementary Figure 8), the comparison between ChaD and non-specific peptides (Figure 4e), the dissociation of the Mtb-SecBTA-HigA1 hexamer upon ChaD titration (Figure 5c and Supplementary Figure 9b-f) and the interaction of the Mtb-SecBTA-HigA1 hexamer with DNA (Supplementary Figure 9a).
