# Supplementary figures and images for: Structural insights into chaperone addiction of toxin-antitoxin systems
Source: Nat Commun. 2019 Feb 15;10:782. doi: 10.1038/s41467-019-08747-4 (PMC6377645; doi:10.1038/s41467-019-08747-4)

## Slide 1
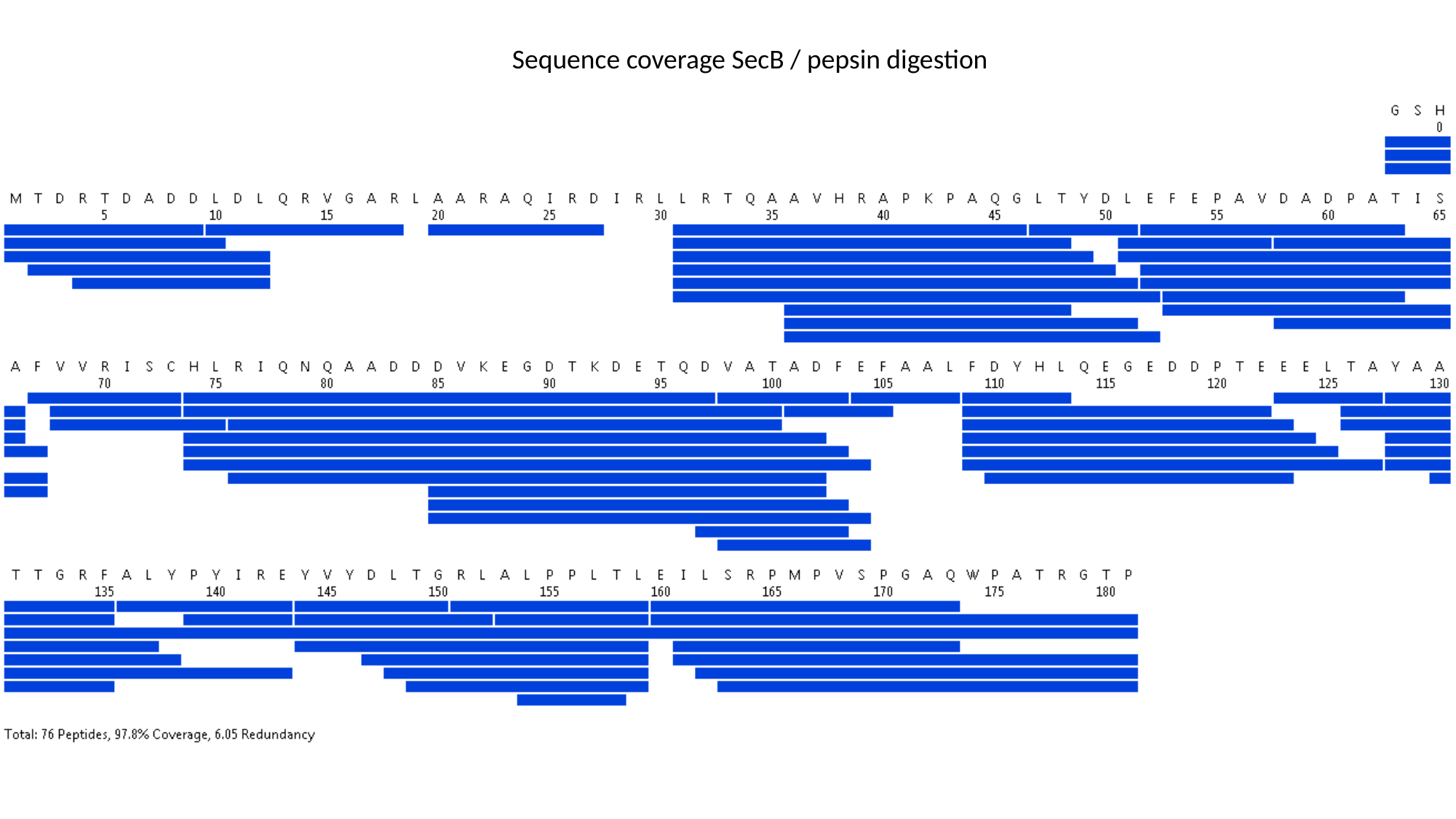

Sequence coverage SecB / pepsin digestion

## Slide 2
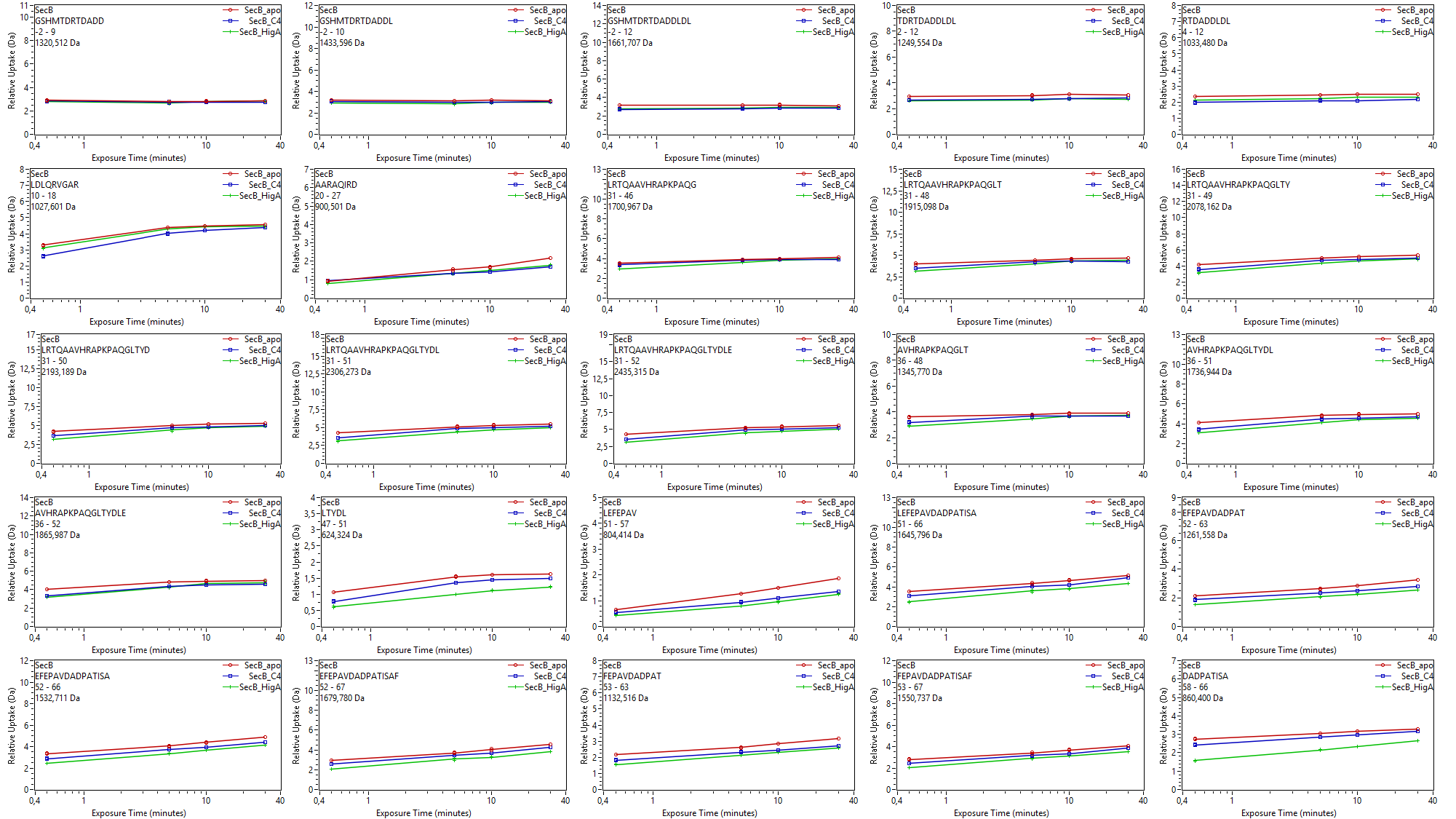

## Slide 3
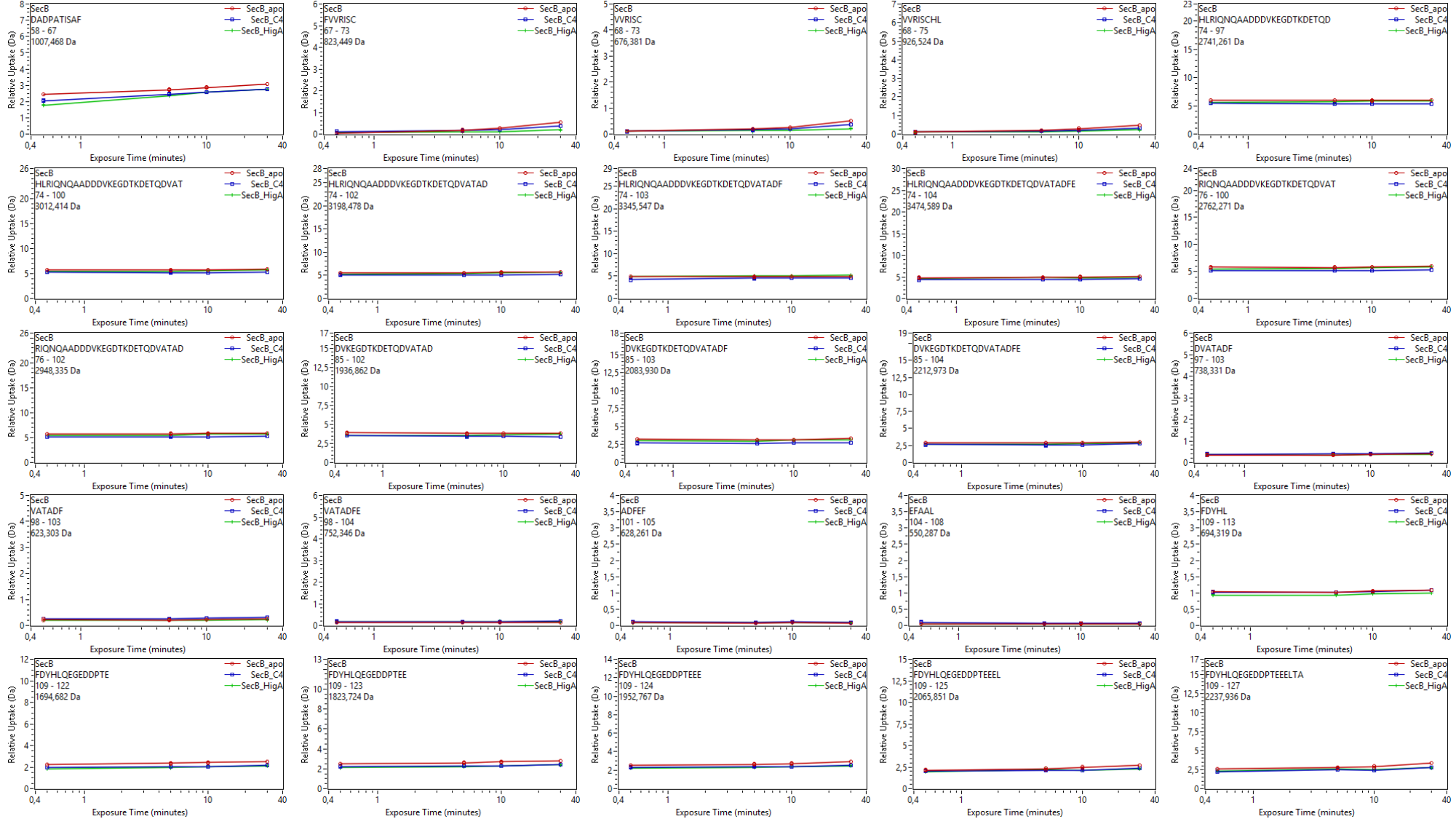

## Slide 4
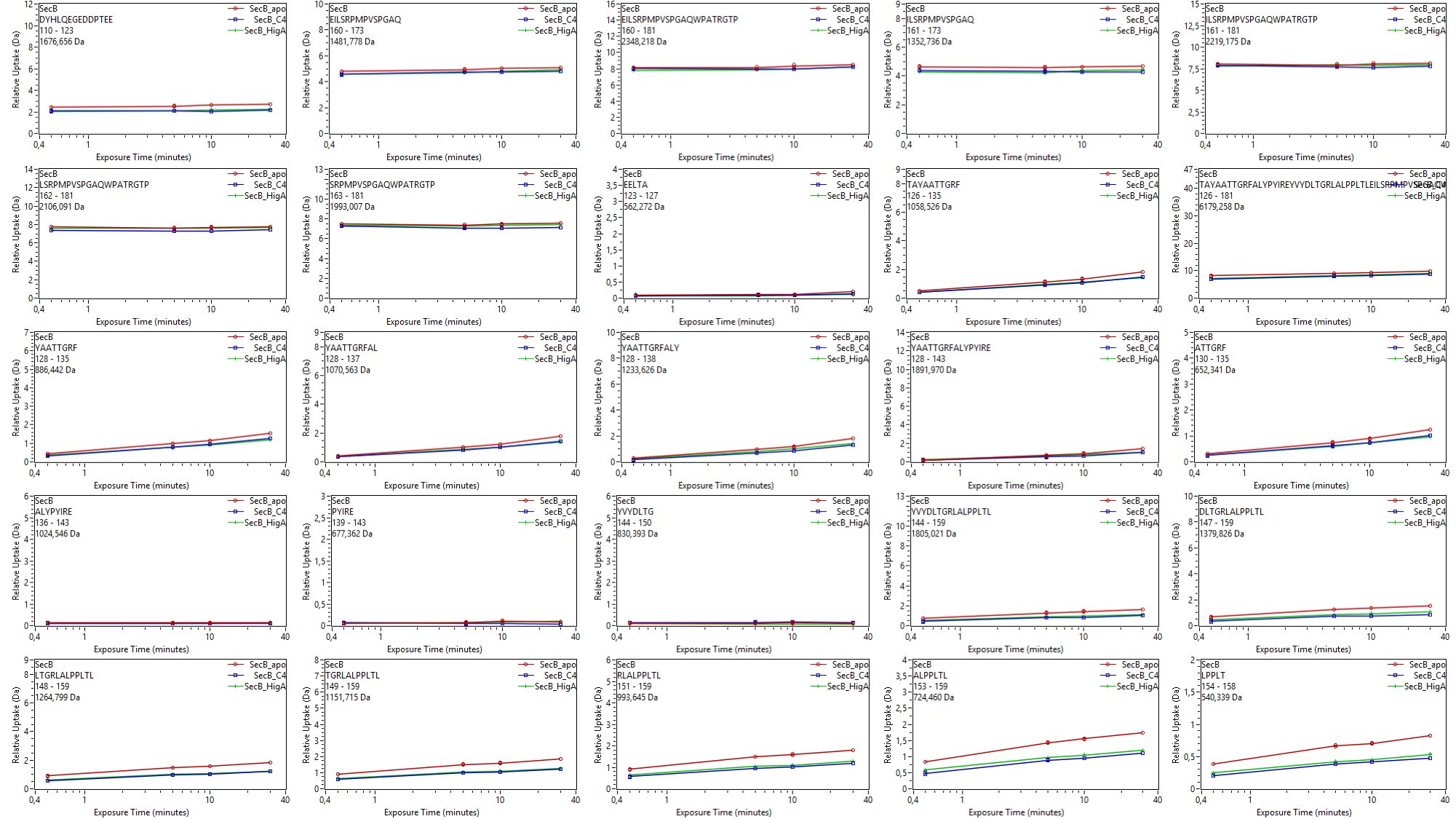

Supplement: Supplementary file 4 — Supplementary Data 1 [file 41467_2019_8747_MOESM4_ESM.zip › HDXMS-Data/Kinetics.pptx]
