# Supplementary material for: Structural insights into chaperone addiction of toxin-antitoxin systems
Source: Nat Commun. 2019 Feb 15;10:782. doi: 10.1038/s41467-019-08747-4 (PMC6377645; doi:10.1038/s41467-019-08747-4)
Supplement: Supplementary file 6 — Reporting Summary [file 41467_2019_8747_MOESM6_ESM.pdf]

naturereseach

Corresponding author(s): Lionel Mourey

Reporting Summary

Nature Research wishes to improve the reproducibility of the work that we publish. This form provides structure for consistency and transparency in reporting. For further information on Nature Research policies, see Authors & Referees and the Editorial Policy Checklist.

Statistical parameters

When statistical analyses are reported, confirm that the following items are present in the relevant location (e.g. figure legend, table legend, main text, or Methods section).

n/a	Confirmed	
☒	☐	The exact sample size (n) for each experimental group/condition, given as a discrete number and unit of measurement
☐	☒	An indication of whether measurements were taken from distinct samples or whether the same sample was measured repeatedly
☒	☐	The statistical test(s) used AND whether they are one- or two-sided<br>*Only common tests should be described solely by name; describe more complex techniques in the Methods section.*
☒	☐	A description of all covariates tested
☒	☐	A description of any assumptions or corrections, such as tests of normality and adjustment for multiple comparisons
☒	☐	A full description of the statistics including central tendency (e.g. means) or other basic estimates (e.g. regression coefficient) AND variation (e.g. standard deviation) or associated estimates of uncertainty (e.g. confidence intervals)
☒	☐	For null hypothesis testing, the test statistic (e.g. F, t, r) with confidence intervals, effect sizes, degrees of freedom and P value noted<br>*Give P values as exact values whenever suitable.*
☒	☐	For Bayesian analysis, information on the choice of priors and Markov chain Monte Carlo settings
☒	☐	For hierarchical and complex designs, identification of the appropriate level for tests and full reporting of outcomes
☒	☐	Estimates of effect sizes (e.g. Cohen's d, Pearson's r), indicating how they were calculated
☐	☒	Clearly defined error bars<br>*State explicitly what error bars represent (e.g. SD, SE, CI)*

Our web collection on statistics for biologists may be useful.

Software and code

Policy information about availability of computer code

Data collection	EDNA for diffraction data collection at the European Synchrotron Radiation Facility<br>MassLynx 4.1 for raw native MS data acquisition<br>HDxDirector 1.0.3.9 for automation of HDX sample preparation
Data analysis	XDS AUTOPROC 1.1.7 for diffraction data integration<br>XSCALE AUTOPROC 1.1.7 for diffraction data scaling software<br>CCP4 software suite 6.2 including PHASER 2.7.0 for molecular replacement, Buccaneer 1.5.1 for initial model building, and REFMAC 5.8.0049 for refinement<br>PDBePISA 1.48 from the EMBL-EBI server for calculating molecular interfaces<br>PyMOL 2.0 with python 2.7 for molecular vizualisation and producing some of the figures<br>ESPript 3 for generating sequence alignment<br>Dali 4 for structural superposition<br>Amber14 for molecular dynamics calculations<br>ASTRA 6.0.2.9 for SEC-MALS analysis<br>ATSAS suite of programs (2.8.4) for SAXS data analysis including PRIMUS 3.4 for SAXS data manipulation<br>NanoTemper analysis software 2.1.2 for MST data<br>MicroCal Origin 5.0 for ITC data<br>UniDec 1.2.6 for deconvolution of mass spectra and Kd estimation

ProteinLynx Global 3.0.2 server for peptide identification
DynamX 3.0 for HDX data analysis

For manuscripts utilizing custom algorithms or software that are central to the research but not yet described in published literature, software must be made available to editors/reviewers upon request. We strongly encourage code deposition in a community repository (e.g. GitHub). See the Nature Research guidelines for submitting code & software for further information.

Data

Policy information about availability of data

All manuscripts must include a data availability statement. This statement should provide the following information, where applicable:

- Accession codes, unique identifiers, or web links for publicly available datasets
- A list of figures that have associated raw data
- A description of any restrictions on data availability

The data that support the findings of this study are available from the corresponding authors on request. The native MS and HDX-MS data are available assupplementary material.
The atomic coordinates and structure factors (code 5MTW) have been deposited in the Protein Data Bank, Research Collaboratory for Structural Bioinformatics, Rutgers University, New Brunswick, NJ (http://www.rcsb.org/).

Field-specific reporting

Please select the best fit for your research. If you are not sure, read the appropriate sections before making your selection.

☒ Life sciences ☐ Behavioural & social sciences ☐ Ecological, evolutionary & environmental sciences

For a reference copy of the document with all sections, see nature.com/authors/policies/ReportingSummary-flat.pdf

Life sciences study design

All studies must disclose on these points even when the disclosure is negative.

Sample size	n/a
Data exclusions	No data exclusions
Replication	Several measurements were performed whenever necessary and they indicated good reproducibility of the corresponding experiments.
Randomization	n/a
Blinding	Blinding was not relevant to our study. All experiments performed rely on objective criteria (diffraction images, spectra, gels, etc.).

Reporting for specific materials, systems and methods

Materials & experimental systems

n/a	Involved in the study	
☐	☒	Unique biological materials
☐	☒	Antibodies
☒	☐	Eukaryotic cell lines
☒	☐	Palaeontology
☒	☐	Animals and other organisms
☒	☐	Human research participants

Methods

n/a	Involved in the study	
☒	☐	ChIP-seq
☒	☐	Flow cytometry
☒	☐	MRI-based neuroimaging

Unique biological materials

Policy information about availability of materials

Obtaining unique materials	All the unique used materials are readily available from the authors.

Antibodies

Antibodies used	rabbit anti-MtbSecBTA antibody
Validation	Validated against MtbSecBTA expressed in Escherichia coli (Bordes et al, Proc Natl Acad Sci U S A. 2011 May 17;108(20):8438-43. doi: 10.1073/pnas.1101189108.PMID:21536872)
